# The role of Rho GTPases’ substrates Rac and Cdc42 in osteoclastogenesis and relevant natural medicinal products study

**DOI:** 10.1042/BSR20200407

**Published:** 2020-07-15

**Authors:** Yuan Liu, Yusheng Dou, Liang Yan, Xiaobin Yang, Baorong He, Lingbo Kong, Wanli Smith

**Affiliations:** 1 Department of Spine Surgery, Honghui Hospital, School of Medicine, Xi’an Jiaotong University, Xi'an, China; 2 Department of Orthopedics, Yan’an University Medical School, Yan’an, China; 3 Department of Shoulder and Elbow Joint, Honghui Hospital, School of Medicine, Xi’an Jiaotong University, China; 4 Department of Psychiatry and Behavioral Sciences, School of Medicine, Johns Hopkins University, Baltimore, MD, U.S.A.

**Keywords:** bone, Cdc42, natural compounds, Rac1, Rac, Rho GTPase

## Abstract

Recently, Rho GTPases substrates include Rac (Rac1 and Rac2) and Cdc42 that have been reported to exert multiple cellular functions in osteoclasts, the most prominent of which includes regulating the dynamic actin cytoskeleton rearrangements. In addition, natural products and their molecular frameworks have a long tradition as valuable starting points for medicinal chemistry and drug discovery. Although currently, there are reports about the natural product, which could play a therapeutic role in bone loss diseases (osteoporosis and osteolysis) through the regulation of Rac1/2 and Cdc42 during osteoclasts cytoskeletal structuring. There have been several excellent studies for exploring the therapeutic potentials of various natural products for their role in inhibiting cancer cells migration and function via regulating the Rac1/2 and Cdc42. Herein in this review, we try to focus on recent advancement studies for extensively understanding the role of Rho GTPases substrates Rac1, Rac2 and Cdc42 in osteoclastogenesis, as well as therapeutic potentials of natural medicinal products for their properties on the regulation of Rac1, and/or Rac2 and Cdc42, which is in order to inspire drug discovery in regulating osteoclastogenesis.

## Introduction

**Figure 1:**
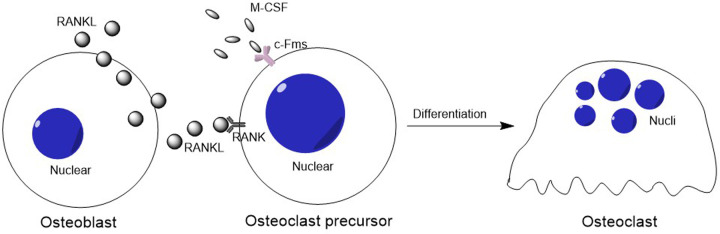
The schematic of osteoclastogenesis The cytokines M-CSF and RANKL (from osteoblasts) bind to its receptors cFms and RANKL present in osteoclast precursors, respectively. Then the M-CSF stimulates osteoclast precursors proliferation and inhibits their apoptosis. Besides that, RANKL interacts with its receptor RANK in osteoclast precursor cells, then osteoclastognesis is induced.

Osteoclastogenesis has been defined as a multistep processes of osteoclast differentiation [1], including several osteoclastic cellular biological functions; such as: migration, cellular contact, cellular fusion and cellular response extracellular factors [2]. Documented studies demonstrated that osteoclastogenesis initially mediated by two critical cytokines, the macrophage colony stimulating factor-1 (M-CSF) and the receptor activator of nuclear factor-κB ligand (RANKL) [3]. M-CSF binds to its receptor (cFms) present in osteoclast precursors, which stimulates their proliferation and inhibits their apoptosis; while, RANKL interacts with its receptor RANK in osteoclast precursor cells, and osteoclastognesis is induced [4] (Figure 1).

However, at the late stage of osteoclastogenesis, osteoclastic polarization characterized the final maturation of bone resorptive osteoclasts. Notably, during the bone resorption process, osteoclastic polarization involves rearrangement of the actin cytoskeleton, in which a filamentous (F)-actin ring that comprises dense continuous zones of highly dynamic podosomes are formed and consequently an area of membrane that develops into the ruffled border is isolated [5,6].

### Cytoskeletal rearrangement during osteoclastogenesis

It is worthy to note that during the cytoskeletal rearrangement in the osteoclastogenesis, podosome is the most prominent cytoskeletal structure for the degradation of mineralized bone matrix and associates with the mobility of osteoclasts [7]. In fact, podosome is not the exclusive organelle in osteoclast, which also includes endothelial cells, and cells from the monocytic lineage such as: dendritic cells (DCs) and macrophages [8]. Regardless, the presentation of podosomes in various cells, podosomes patterning plays a crucial and unique role in the support osteoclast final maturation [8]. As early as individual podosome forms within an osteoclast, they are collectively and sequentially organized into different patterns along the life of the same cell. However, these patterns evolve along with osteoclastogenesis from monocytes/macrophages to osteoclast precursors, further to the bone resorptive matured osteoclast. In the early stage of osteoclastogenesis, podosome pattern from apparently random groups of ‘clusters’ to circle pattern ‘rings’ in the middle-term stage [9]. Eventually, in the late stage of osteoclastogenesis, podosome patterns into much massive circular structures, i.e., either ‘sealing zone like structures’ (SZL, also known as ‘belts’) or ‘sealing zones’ (SZ) [10].

### Rac isoforms (Rac1 and Rac2) in regulation of cytoskeletal arrangement during osteoclastogenesis

It has been reported that Rac1 and Rac2 are critical GTPases for osteoclast formation and maturation. In fact, Rac1 and Rac2, are intimately associated with the organization of the different types of cellular cytoskeleton, such as: osteoclasts, DCs and macrophages. Notably, these two isoforms are also involved in the osteoclastic adhesive function formation and subsequent bone resorption [11,12]. However, the specific role of Rac1 and Rac2 in osteoclastogenesis is still unknown. For example, osteoclasts contain NADPH diaphorase activity [13,14], and free radicals which both could influence bone resorption, however, Rac1 and Rac2 are also essential components of NADPH oxidase [15–18], the enzyme responsible for generating free radicals. Besides that, a study has also demonstrated that Rac1 and Rac2 could regulate the generation of reactive oxygen species (ROS) [19] and actin remodeling participating in the osteoclastogenesis regulation. Recent study has found that both Rac1 and Rac2 are required for normal RANKL-induced osteoclast differentiation, but Rac1 deletion results in a more profound reduction in osteoclast formation *in vitro* because of its regulatory role in pre‐osteoclast M‐CSF mediated chemotaxis and actin assembly and RANKL‐mediated ROS generation [20]. These results speculated that Rac1 and Rac2 might function in osteoclastic organelle actin dynamics regulating, such as: actin filament ends and podosomes. In fact, Rac1 and Rac2 proteins have overlapping roles in podosome assembly and SZL formation by localizing Arp2/3 at podosome sites during osteoclastogenesis [7,21–27]. Osteoclasts generated from the Rac1 and Rac2 double knockout mouse are devoid of podosomes and SZ, which finally showed impaired bone resorption capacities [24,28–32]. Notably, however, these defects are observed only if Rac1 and Rac2 deletion occurs at the early osteoclast precursor stage, which means the Rac1- and Rac2-deficient osteoclasts lack the capabilities of actin cytoskeletal formation.

### The role of Cdc42 in regulation of the podosome of osteoclast

Cdc42 is another Rho family small GTPase [33]. As a downstream signaling of RANKL, Cdc42 might interact with the Crib domain of the adaptor Par3 [34,35], Par6 and atypical PKC (aPKC) [36–38], which forms a quaternary complex to cascade the upper signaling transduction from RANKL and RANK binding, further stimulating the osteoclastogenesis. However, unlike Rac1 and Rac2 the definition role of Cdc42 in osteoclastogenesis is much clearly associated with its actin regulative effects, i.e. the podosome regulation. Recent studies using mice with increased Cdc42 activation due to knockout of its negative regulator Cdc42GAP have shown increased SZ formation and bone resorption, compared with wildtype cells [27,39].

Cdc42 stands as a central player in the regulation of podosome dynamics as it orchestrates podosome actin polymerization, which from the monomeric globular (G)-actin into filamentous (F)-actin, through its canonical effector, Wiscott–Aldrich Syndrome protein (WASp) [40–42]. WASp depletion in macrophages leads to a virtual absence of podosomes and a defective chemotactic response under a gradient of M-CSF. Cdc42 binds directly to WASp, a multidomain adapter protein regulating transmission of signals to the actin cytoskeleton. This binding, together with phosphorylation of WASp on tyrosine, induces a dramatic conformational change [40,41,43]. The hydrophobic core is disrupted, releasing the VCA (Verprolin Homology domain-cofilin homology domain–acidic region) domain and enabling its interaction with the Arp2/3 complex, thereby promoting actin nucleation [44–46].

### Natural products targeting the regulation of Rac1 and Rac2, and Cdc42

**Figure 2:**
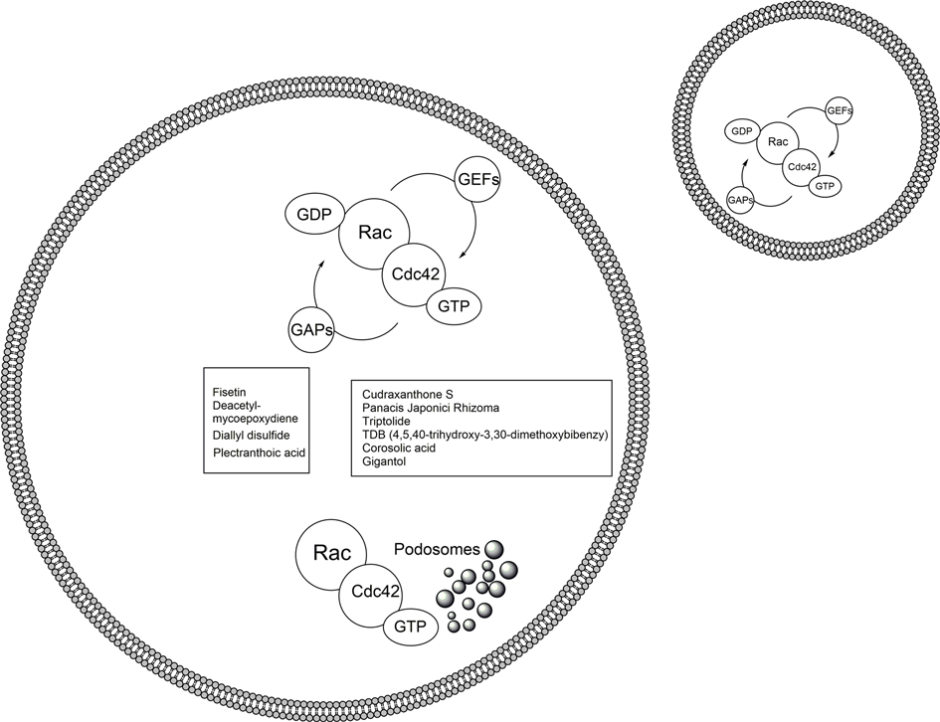
The schematic of molecular mechanisms of Rho GTPases Rac and Cdc42, and relevant therapeutic natural compounds During the osteoclastogenesis, after RANKL and RANK binding, the intracellular Rac1, Rac2 and Cdc42 are via GTP associate with podosomes regulation. However, these regulation effects might inhibited by various compounds (Left panel: inhibitory compounds for Rac1 and Rac2; Right panel: inhibitory compounds for Cdc42).

Natural products and their molecular frameworks have a long tradition as valuable starting points for medicinal chemistry and drug discovery. Recently, there has been a revitalization of interest in the inclusion of these chemotypes in compound collections for screening and achieving selective target modulation. Although currently, there have been no reports on the natural product, which could play a therapeutic role in bone loss diseases (osteoporosis and osteolysis) through the regulation of Rac1/2 and Cdc42 during osteoclasts cytoskeletal structuring. There have been several excellent studies exploring the therapeutic potentials of various natural products in regulating cancer cells migration and function (Table 1). Here we collected several natural products with a focus on recent advances in their properties on the regulation of Rac1 and/or Rac2 and Cdc42, and related signaling molecules, in order to inspire drug discovery in regulating osteoclastogenesis (Figure 2).

**Table 1:** The source, structure, cells or animal models and mechanisms of ten natural compounds

Compound name	Source	Structure	Cell lines used for *in vitro* studies	Animal models	Dose	Mechanisms	Studies
Fisetin	*polyphenolic molecule of flavonoids*	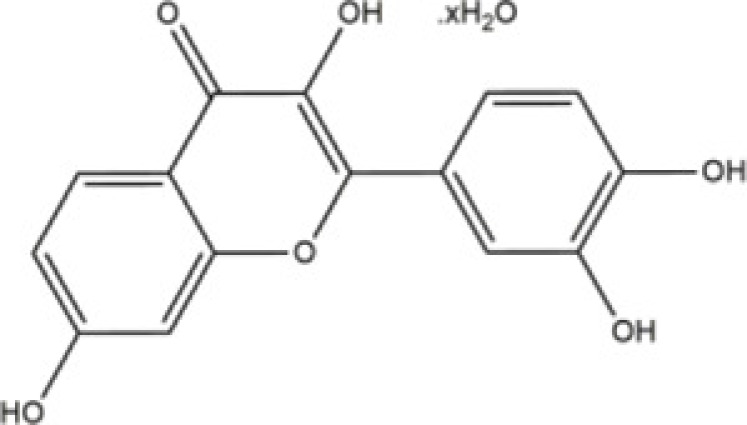	Neuron	Wistar rats	30 mg/kg	Rac1/Cdc42	Jacob [50]
Deacetyl- mycoepoxydiene	*Phomopsis sp., of costal mangrove*	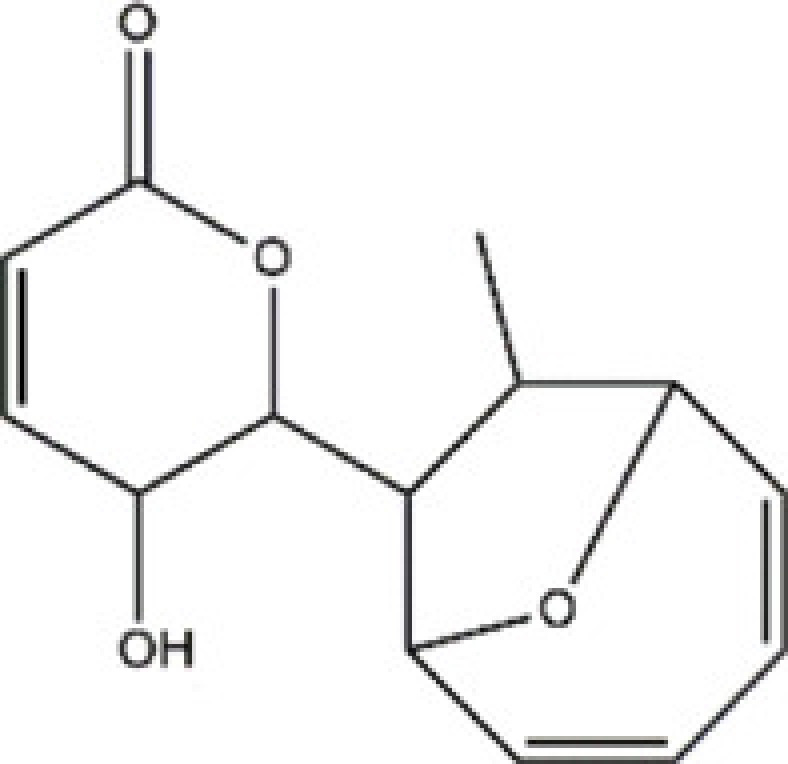	Human breast cancer MCF-7 cells	BABL/c mice	5, 10, 20 mg/kg	Rac1	Zhao [59]
Diallyl disulfide	*garlic*	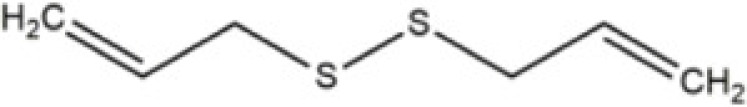	Human gastric cancer MGC803 cell line	BALB/c nude mice	100mg/kg	Rac1	Su [55]
Plectranthoic acid	*Ficus microcarpa*	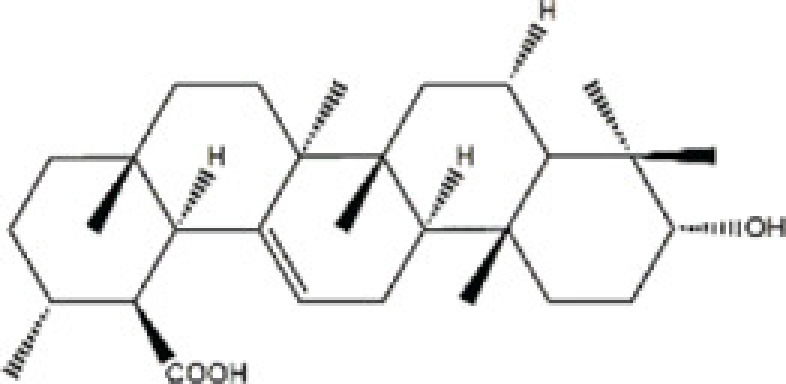	Prostate cancer cell lines (DU145, PC3, NA22, NB26)	N/A	N/A	Rac1	Akhtar [61]
Cudraxanthone S	*Cudrania cochinchinensis*	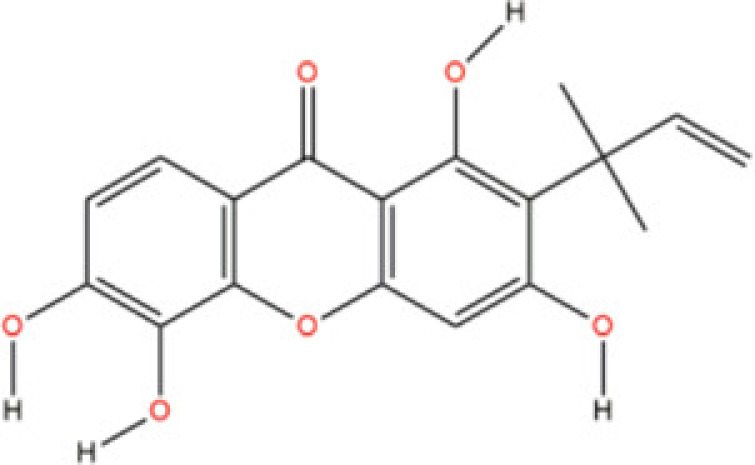	N/A	N/A	N/A	Cdc42	Gopal [65]
Panacis Japonici Rhizoma	*Panax japonicus C. A. Meyer*	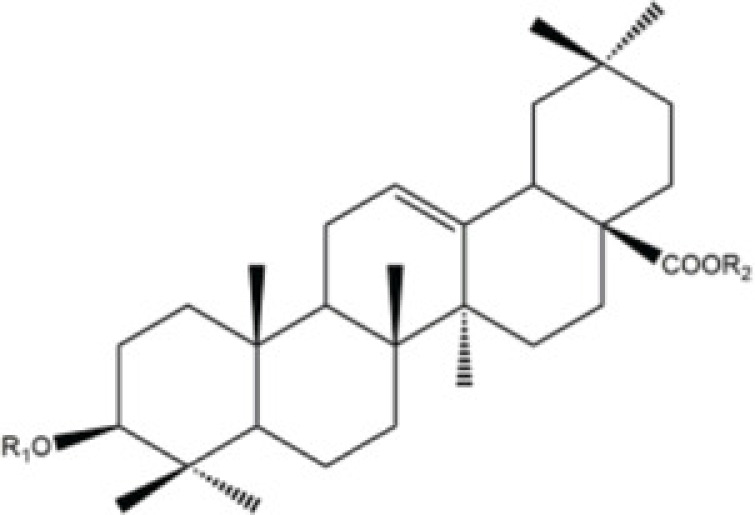	A2780 cell line	N/A	N/A	Cdc42	Chen [66]
Triptolide	*Triterygium wilfordii Hook. f.*	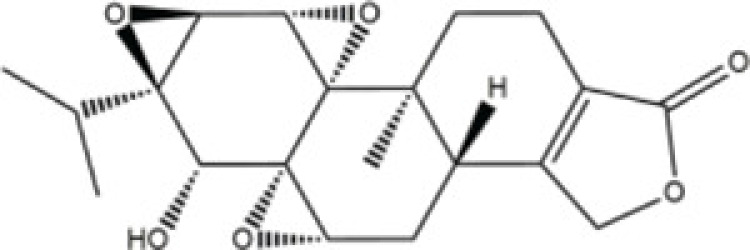	Sprague–Dawley (SD) rats	Sprague–Dawley (SD) rats	100 mg/kg	Rac1, Cdc42	Wang [51]
TDB (4,5,40-trihydroxy-3, 30- dimethoxybibenzy)	*Dendrobium ellipsophyllum Tang and Wang*	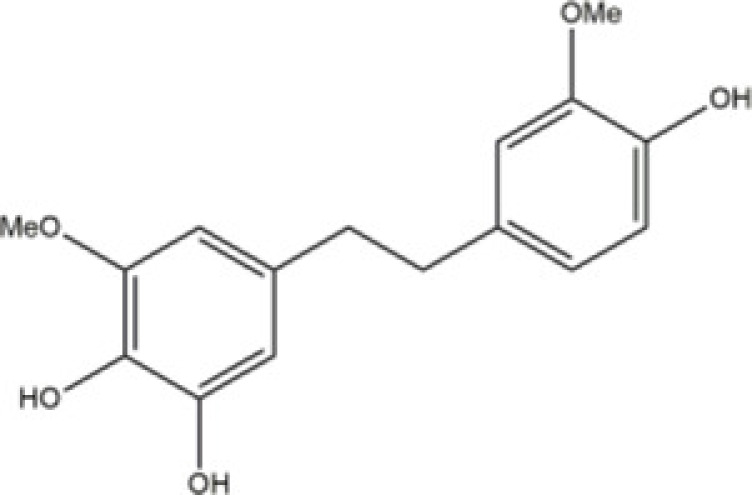	Human lung cancer H292 cells	N/A	N/A	Rac1/Cdc42	Chaotham [74]
Corosolic acid	*Actinidia chinensis*,	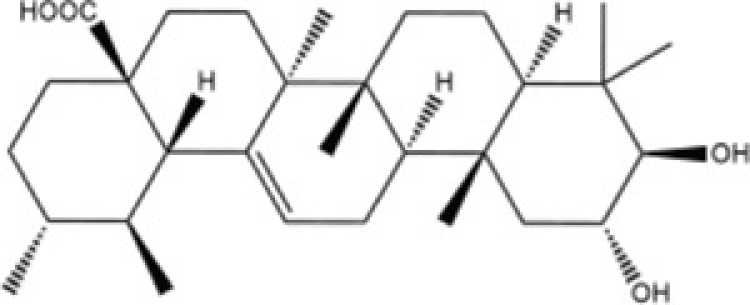	Hepatocellular carcinoma cell lines (Huh7, HepG2 and Hep3B)	NOD/SCID mice	5 mg/kg	Cdc42	Ku [77]
Gigantol	*Thai orchid, Dendrobium draconis*	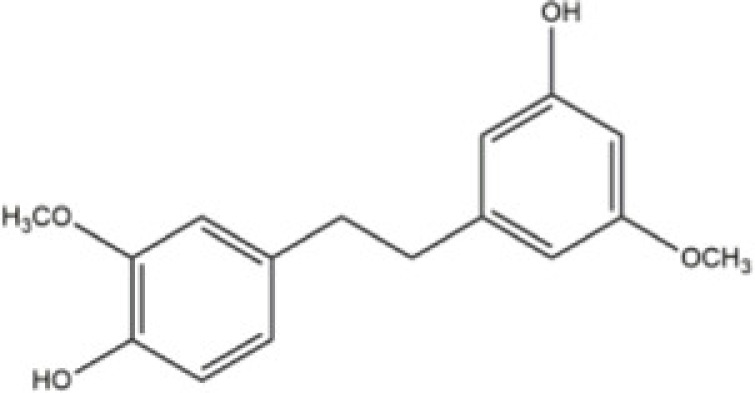	Human lung carcinoma cells NCI-H460 and NCI-H292	N/A	N/A	Cdc42	Charoenrungruang [78]

### Rac1 and Rac2 regulative natural products

*Fisetin* (*3,3′,4′,7-Tetrahydroxyflavone*), is natural product that could be found in vegetables and fruits [47]. *Fisetin* has been well established and possesses antioxidant [48] and anti-neurodegenerative progression [49]. Most recently, Jacob et al. [50] have reported *Fisetin* showed a significant protective effect on developmental Methyl mercury neurotoxicity in the F_1_ generation of MeHg exposed rats. In that, Methyl mercury is a teratogenic and neurodevelopmental toxicant in the environment. Whereas MeHg could affect several biological pathways critical for brain development. Most recently, authors present study validated the effect of *Fisetin* on developmental MeHg exposure induced alterations in mitochondrial apoptotic pathway and Rho GTPase mRNA expressions in hippocampus of F_1_ generation rats. Their extensive study showed that Fisetin against gestational MeHg exposure induced changes in expression of ERK/Caspase 3 genes of apoptosis signaling pathway and Rho A/Rac1/Cdc42 genes of Rho GTPase signaling pathway in hippocampus of F_1_ generation weaning Wistar rats.

*Deacetyl-mycoepoxydiene* (DA-MED) is a 248 molecular weight compound that has been isolated from the endophytic fungus, *Phomopsis sp.*, of *costal mangrove* plants and has been shown to be a secondary metabolite with a rare oxygen-bridged cyclopentadiene skeleton [51]. This compound has cytotoxic activities toward various cell lines, including A549, HCC-S102 and HepG2 cells with IC_50_ values ranging from 1.05 to 1.95 mg/ml. Recently, Xie et al. [52] have reported that DA-MED treatment drives Rac1 activation and promotes robust production of ROS, activating mitochondrial permeability transition and the intrinsic apoptotic pathway. Knockdown of Rac1 decreases ROS production in DA-MED-treated cells, resulting in a concomitant decrease in DA-MED-induced apoptosis. DA-MED-activated Rac1 induces autophagy by inhibiting mammalian target of rapamycin, leading to anti-apoptotic and anti-metastatic effects. Therefore, the present study provides novel insight into the complex cytotoxic and pro-survival mechanisms associated with a potent Rac1 agonist and suggests that further development of more potent Rac1 agonists could be an effective strategy for future non-small cell lung cancer treatments.

*Diallyl disulfide* (DADS), one of the sulfur compounds derived from garlic, exhibits biological activity via modulating molecules and signaling pathways in various cell physiologies [53–57]. These properties suggesting that DADS could be used as a potential therapeutic compound for the treatment or prevention of various diseases. Moreover, study has demonstrated that transforming growth factor-β1 (TGF-β1) could promote epithelial–mesenchymal transition (EMT), invasion and proliferation through the activation of Rac1 and β-catenin signaling pathways. Therefore, Su et al. [55] have conducted a study for investigating the effects of DADS on TGF-β1-induced EMT and cellular invasion. Primarily, they found TGF-β1 treatment augmented EMT and invasion, concomitantly with increased expression of Rac1 and β-catenin. However, the DADS treatment could decrease the activities of Rac1 and β-catenin. DADS, TGF-β1 receptor inhibitor as well as Rac1 inhibitor antagonized the up-regulation of the TGF-β1-induced expression of these genes, abolishing the enhanced effects of TGF-β1 on EMT and invasion. These data indicated that the blockage of TGF-β1/Rac1 signaling by DADS may be responsible for the suppression of EMT and cellular invasion.

Mulberry (*Morus alba L.*) is a common fruit in temperate, subtropical and tropical areas, and contains abundant polyphenols and anthocyanin components [58,59]. Study showed the anthocyanins from the mulberry could inhibit the B16-F1 cell linage invasion [60]. The underlying molecular mechanisms is anthocyanins partly suppressed the Ras/PI3K signaling pathway. In addition, mulberry polyphenol extract (MPE) is rich in polyphenols that have antioxidant, anti-inflammatory, anti-aging, anti-obesity and anti-tumor effects. Considering the biological effects of anthocyanins, further study performed by Yu et al. [58] investigated that MPE on treating vascular smooth muscle cellular migration and proliferation. Their results showed that MPE could suppress the expression of FAK, Src, PI3K, Akt, c-Raf, and inhibit the signaling axis of FAK/Src/PI3K in cell. Besides that, their study also showed that MPE decrease the expression of small Rac1 and Cdc42 to affect F-actin cytoskeleton rearrangement.

As aforementioned, cytoskeletal structure rearrangement grant various cellular functions in various cell linages, such as: podosome patterning in osteoclasts and EMT transition. This has led to a surge in the efforts for identification of safer and more effective compounds which can modulate these cellular behaviors. *Plectranthoic acid* (PA), a natural compound isolated from the extracts of *Ficus microcarpa*, has been reported to possess the capability to induce cell cycle arrest and apoptosis in prostate cancer cells [61,62]. Recently, Akhtar et al. [61] extensively studied the PA biological effects on suppressing the cellular migration. Through the proteomic analysis, authors identified that Rac1 is the major cadherin signaling protein modulated with PA treatment.

### Cdc42 regulative natural products

*Cudrania cochinchinensis* (Moraceae) has been reported for its potent biological activites such as: anti-inflammation [63] and neuroprotective effects [64]. Whereas, the compound *Cudraxanthone-S* derived from *C. cochinchinensis* was studied for its pharmacokinetics and binding potential in treating the fungal infection of *Candida albicans*, which could cause several lethal infections in immune-suppressed patients and recently emerged as drug-resistant pathogens worldwide [65]. Authors found that *Cudraxanthone-S* had exhibited ability on regulating the Cdc42 in MAPK signaling pathway.

*Panacis Japonici Rhizoma* (PJR), derived from dry rhizome of *Panax japonicus C. A. Meyer* (Araliaceae), distributes in the southwest of China [66–68]. As a widely used focal medicine, the PJR manifested similar clinical merits in anti-tussive and anti-inflammatory diseases [69,70]. Recently, Chen et al. [71] have demonstrated that PJR could suppress the HEY and A2780 cells migration and invasion by decreasing the Cdc42 and Rac activities.

*Triptolide* (TP), derived from the medicinal plant *Triterygium wilfordii Hook. f.* (TWHF), is a diterpene triepoxide with variety of biological and pharmacological activities [72]. Wang et al. [73] has studied the cytoskeletal structuring effects of TP on Sertoli cells, which play a critical role during spermatogenesis. Their study results demonstrate that TP can regulating the Sertoli cellular cytoskeleton structuring via inhibiting the expression of Cdc42.

The compound (*4,5,4′-trihydroxy-3,3′-dimethoxybibenzyl* (TDB)) extract from *Dendrobium ellipsophyllum Tang and Wang*, has been demonstrated to have antimetastatic activity through the sensitization of detachment-induced cell death [74,75]. Study from Chaotham et al. [76] showing that TDB reduced such cell migration and invasion by decreasing migration-regulating proteins, including integrins αv, α4, β1, β3 and β5, as well as downstream signaling proteins, such as activated focal adhesion kinase (pFAK), activated Rac1 and Cdc42. As the presence of cellular protrusion, called filopodia, has been indicated as a hallmark of migrating cells, we showed that the reduction in the mentioned proteins correlated well with the disappearance of filopodia. In summary, the present study demonstrates the promising activity of TDB and its mechanism in the inhibition of lung cancer cell migration, which might be useful for encouraging the development of this compound for anti-metastatic approaches.

Inhibition of VEGFR2 activity has been proposed as an important strategy for the clinical treatment of hepatocellular carcinoma (HCC). *Corosolic acid* (CA), which exists in the root of *Actinidia chinensis*, as having a significant anti-cancer effect on HCC cells by decreasing the tumor cellular migration. Ku et al. [77] have extensively studied the effects of CA on its cellular regulating effects found that CA inhibits VEGFR2 kinase activity by directly interacting with the ATP-binding pocket. Moreover, they found CA could decrease the VEGFR2/Src/FAK/Cdc42 axis, subsequently decreasing F-actin formation and migratory activity *in vitro*.

*Gigantol* is a bibenzyl compound derived from the *Thai orchid, Dendrobium draconis*. It exhibits significant cytotoxic activity against several cancer cell lines. Study conducted by Charoenrungruang et al. [78] demonstrates that gigantol suppresses the migratory cellular behavior via decreasing Cdc42, thereby suppressing filopodia formation. The inhibitory activity of *Gigantol* on lung cancer cellular migration suggests that this compound may be suitable for further development for the treatment of osteoclastogenesis by regulating the osteoclastic cytoskeletal structuring.

## Conclusion

Characterized by the unique property, osteoclasts have been extensively studied for their differentiation and cellular functions during the bone homeostasis and pathological process, which makes them as a critical target for therapy in the bone loss diseases, such as: osteoporosis and osteolysis. Given that the low production costs and the increasing evidence of the ability to target the cellular activities and signaling cascades relevant to various diseases, naturally occurring compounds have received extensive attention as potential therapeutic osteoclastogenesis. Our current review has outlined some naturally occurring compounds, which have shown merit in terms of regulating macrophage polarization. However, given that current natural compounds have the Rac and Cdc42 regulatory effects on cancer cell line, the specific mechanisms and therapeutic effects on osteoclastognesis remain incompletely understood. Clearly, more in-depth characterization of osteoclast cytoskeleton rearrangement and relevant therapeutic compounds should be conducted to identify the best possible strategies.

## Data Availability

All data and materials are included in the manuscript.

## Abbreviations

Arp2/3: actin-related proteins-2/3
CA: corosolic acid
DADS: diallyl disulfide
DA-MED: deacetyl-mycoepoxydiene
DC: dendritic cell
EMT: epithelial–mesenchymal transition
HCC: hepatocellular carcinoma
MPE: mulberry polyphenol extract
M-CSF: macrophage colony stimulating factor-1
PA: plectranthoic acid
PJR: *Panacis Japonici Rhizoma*
PKC: protein kinase C
RANKL: receptor activator of nuclear factor-κB ligand
ROS: reactive oxygen species
SZ: sealing zone
SZL: sealing zone like structure
TDB: 4,5,4′-trihydroxy-3,3′-dimethoxybibenzyl
TGF-β1: transforming growth factor-β1
TP: triptolide
VEGFR: vascular endothelial growth factor receptor
WASp: Wiscott–Aldrich Syndrome protein
